# Pallidal GABA B receptors: involvement in cortex beta dynamics and thalamic reticular nucleus activity

**DOI:** 10.1186/s12576-023-00870-8

**Published:** 2023-06-16

**Authors:** Nelson Villalobos, Victor Manuel Magdaleno‐Madrigal

**Affiliations:** 1 Academia de Fisiología, Escuela Superior de Medicina, Instituto Politécnico Nacional, Plan de San Luis y Díaz Mirón, Colonia Casco de Santo Tomás, 11340 México City, México; 2 Sección de Estudios de Posgrado e Investigación de la Escuela Superior de Medicina, Instituto Politécnico Nacional, Plan de San Luis y Díaz Mirón, Colonia Casco de Santo Tomás, 11340 Mexico City, Mexico; 3 Laboratorio de Neuromodulación Experimental, Dirección de Investigaciones en Neurociencias, Instituto Nacional de Psiquiatría Ramón de la Fuente Muñiz, Mexico City, Mexico; 4 Carrera de Psicología, Facultad de Estudios Superiores Zaragoza-UNAM, México City, México

**Keywords:** Globus pallidus, Thalamic reticular nucleus, Tonic inhibition, Beta band, Desynchronization, GABA B receptor, Network, Disinhibition

## Abstract

The external globus pallidus (GP) firing rate synchronizes the basal ganglia-thalamus-cortex network controlling GABAergic output to different nuclei. In this context, two findings are significant: the activity and GABAergic transmission of the GP modulated by GABA B receptors and the presence of the GP-thalamic reticular nucleus (RTn) pathway, the functionality of which is unknown. The functional participation of GABA B receptors through this network in cortical dynamics is feasible because the RTn controls transmission between the thalamus and cortex. To analyze this hypothesis, we used single-unit recordings of RTn neurons and electroencephalograms of the motor cortex (MCx) before and after GP injection of the GABA B agonist baclofen and the antagonist saclofen in anesthetized rats. We found that GABA B agonists increase the spiking rate of the RTn and that this response decreases the spectral density of beta frequency bands in the MCx. Additionally, injections of GABA B antagonists decreased the firing activity of the RTn and reversed the effects in the power spectra of beta frequency bands in the MCx. Our results proved that the GP modulates cortical oscillation dynamics through the GP-RTn network via tonic modulation of RTn activity.

## Introduction

The networks formed by the external globus pallidus (GP) through its afferents allow it to function as a hub that modulates the flow of information in the basal ganglia (BG)-thalamus (Th)-cortex (Cx) network [1–3]. The modulation of network activity is mediated by the GP spiking rate [1]. Alterations in both the firing pattern and oscillatory dynamics in the frequency beta range of the GP are of paramount importance in BG function and dysfunction [4–9]. In this framework, the GP sends GABAergic axons to the motor region of the thalamic reticular nucleus (RTn) [10, 11].

The RTn is a master huddle of neurons that link up network dynamics based on its firing activity [12–14]. In this way, RTn neurons shape the thalamic output to the Cx by GABAergic synapses with thalamocortical (TC) and corticothalamic (CT) axons [15, 16]. The firing activity is modulated by both inputs and neuromodulators [17, 18] and is the base that originates both physiological [19, 20] and pathological oscillations [20–22]. However, the participation of the RTn in motor control has yet not been established with the exception of an interesting report showing the participation of the RTn in locomotor activity [23].

In motor physiology, the BG modulates the motor sector of the Th by two output trails (the internal GP and substantia nigra pars reticulata) and relays the motor signal to the Cx [24, 25]. In the above setting, the current model of the BG-Th-Cx network does not consider the presence and contribution of the GP–RTn pathway. However, substantial evidence shows a morphological and functional connection between the neurons of the motor sector of the RTn and the motor nuclei of the Th [26].

GABAergic systems reconfigure oscillatory brain dynamics by metabotropic GABA B receptors (GABA B-Rs). In the synapse, these systems participate by modulating transmission in two ways: they inhibit neurotransmission at the presynaptic level by inhibiting voltage-gated Ca^+2^ channels, and at the postsynaptic level, they modulate G-protein-coupled inward-rectifying K^+^ channels and voltage-gated Ca^+2^ channels in the somatodendritic region [27]. At the neuronal network level, presynaptic GABA B-Rs at excitatory and inhibitory synapses induce inhibitory and disinhibitory effects, respectively [28, 29]. Additionally, presynaptic GABA B-Rs mediate tonic inhibition [30–32]. In the GP, both the expression of GABA B-Rs and their functional implication in the firing frequency are currently accepted [33–38], but their involvement in GP targets has been little explored.

In the context of the BG-Th-Cx circuit, the following evidence is essential: an increase in inhibition during phasic transmission by the overflow of GABA to the extrasynaptic area [39]. At the GP level, this event activates presynaptic GABA B-Rs in both the striatopallidal and subthalamic terminals and subsequently lessens GABA and glutamate release, respectively [40]. However, previous evidence has shown that both higher levels of GABA in the GP [41] and the administration of glutamate and GABA modulate the spontaneous firing of the RTn [42]. The firing rates modulate the oscillation dynamics in the global brain network [9, 43–45]. Thus, the functional importance of the firing pattern of both the GP and the RTn in their respective circuits is widely accepted. In addition, the presence of the GP-RTn connection suggests a functional implication in the oscillatory dynamics of the cerebral cortex.

The previous framework allows us to hypothesize that activation of pallidal GABA B-Rs disinhibits RTn neurons and thereby modulates oscillations in the motor cortex (MCx). To test this hypothesis, we used the extracellular unit recording of RTn neurons, pharmacological manipulation of GABA B-Rs into the GP, and electroencephalogram (EEG) of the MCx in anesthetized rats. The results described below suggest that GABA B-Rs of GP participate in the oscillatory dynamics of the MCx in the beta frequency band and thus modulate the RTn via the GP-RTn pathway.

## Experimental protocol

During the experiments, the animals were handled in agreement with the guidelines of the ESM-IPN in accordance with the International Animal Care and Use Committees (IACUCs) and the local Animal Ethics Committee of Instituto Nacional de Psiquiatría Ramón de la Fuente Muñiz. The experimental protocols followed the Norma Official Mexicana for the care and use of laboratory animals (NOM-062-ZOO-1999) and the Guide for Care and Use of Laboratory Animals published by the U.S. National Institute of Health. Efforts were made to minimize the number of animals used and their suffering.

### Subjects and stereotaxic procedure

Male Wistar rats weighing 220–260 g were used for the experimental procedure. The rats were maintained in individual cages in a room with an ambient temperature of 20–24 °C and a 12/12-h light/dark cycle and given free access to water and food.

Prior to the stereotaxic procedure, coordinates were obtained with a rat brain atlas [46]. For ipsilateral implantation of the recording electrode and injection cannula, the surgery was conducted under anesthesia administered through an intraperitoneal injection of 1.25 mg/kg urethane (Sigma–Aldrich). The anesthetized rat was placed in a stereotaxic instrument (David Kopf, Tujunga, CA, USA), set down in a heating pad to conserve the body temperature between 37 and 38 °C and monitored with a rectal thermometer system (Frederick Haer, Bowdoin ME, USA). The craniotomy for the RTn electrode was performed at the following coordinates: 1.4 mm posterior, 1.2–2.1 mm lateral relative to the bregma, and 5.3–7 mm deep relative to the dura. The GP was 0.6 mm posterior and 2.4–3 mm lateral relative to the bregma and 5–7 mm deep relative to the dura. The injection device for pharmacological handling was implanted in the core of the GP at an angle of 60° relative to the horizontal in the lateral plane. The coordinates were 0.8 mm posterior and 5.8 mm lateral to the bregma and 5.8 mm deep into the dura mater. The EEG from the MCx was obtained by implanting steel screws 3.70 mm anterior to the bregma and 1.9 mm lateral to the midline and a grounding electrode above the parietal bone.

### Electrophysiology

Extracellular unit recordings analyzed the RTn or GP firing activity using glass micropipettes filled with 2 M NaCl and a resistance of 5–10 MΩ. The signals were amplified 10,000 x, bandpass filtered between 0.3 and 3 kHz (DAM-80 WPI, Sarasota, FL, USA), and saved to a PC for thorough offline analysis. For data analysis, time segments were defined in the complete recording (these parameters were established according to previous studies [41]; based on the rate of spontaneous activity. Once the neuron showed stable activity, the mean value and standard deviation (SD) of the firing rate during a 120-s segment (1-s bins) before drug administration was calculated and was considered as baseline firing. Changes in firing rate during a 180-s period from 30-s after the end of drug application were examined. The effect of drug application was considered significant if the firing rate exceeded a level of the mean ± 2SD. The duration was defined as the time during the significant change. The mean firing rate during the 180-s period was also calculated and compared with the baseline activity. After setting these parameters, the coefficient of variation (CV) was calculated as the ratio of the standard deviation of the interspike interval (ISI) to the mean ISI. The EEG signals were amplified, bandpass filtered (1–100 Hz), and digitized (300 samples/s). A spectral analysis of the EEG data was performed by fast Fourier transformation from a 5-s epoch ([FFT]; Hanning window function; data point block size of 1024; resolution of 0.9766 Hz). A custom MATLAB script (2020b MathWorks, Natick, MA, USA) was used to develop the spectrograms.

Before analysis, the EEG recording data were digitally filtered (bandpass: 5–50 Hz) in each time window to avoid the existence of artifacts. The power spectral analysis was in the 10–30-Hz range (due to participation of the GP in this frequency range), and the coherence analysis was used to evaluate the activity of the MCx and RTn in the same frequency range. The same parameters were applied to the coherence and power spectral analysis (window, block, and resolution for FFT).

The coherence analysis was based on the following equation:

$$C_{r \, m} \left( { \, f} \right) = \left| { \, P_{r \, m} \left( { \, f} \right) \, } \right|^{2} /\left[ { \, P_{r \, r} \left( { \, f} \right) \, P_{m \, m} \left( { \, f} \right)} \right].$$

where P_r m_ is the cross-power spectral density of two signals, (*r*) corresponds to the signal of the RTn, and (*m*) corresponds to the signal of the MCx. In addition, P_r r_ (*f*) and P_m m_ (*f*) are the power spectral densities of the RTn and MCx, respectively. Thus, the coherence values were between 0 and 1 and are considered significant if the values lie above the confidence level. The offline analysis was accomplished using Spike 2 analysis software (Cambridge Electronic Design, Cambridge, UK).

### Drug application

Before use, baclofen and saclofen (Sigma‒Aldrich) were dissolved in 0.9% w/v NaCl solution. Neurons with stable baseline firing for 5 min were selected for unilateral application into the GP during RTn or pallidal recording. The injection volume for every infusion was 100 nl. A maximum of five applications were administered to each rat, and the distance and interval between applications were at least 1 mm and 35 min. The injection cannula system (30 gauge) was connected to a microsyringe (Hamilton, 10 µl) through a polyethylene tube and to a precision micrometer head. The infusion was performed at a rate of 50 nl/15 s. The doses used in the present study were in the range applied previously to the GP [35, 36, 47].

### Histology

The rapid procedure method [48] was used to confirm the position of the electrode tip and cannula. After receiving a lethal dose of pentobarbital (150 mg/kg, i.p.), the rats were transcardially perfused with 4% formaldehyde. The brains were obtained and sliced at a width of 50 µm. The experiment was omitted when the electrode and cannula were outside the nuclei of interest.

### Statistical analysis

Statistical comparisons were made using OriginPro8 (OriginLab, Northampton, MA, USA). The significance (a value of p < 0.05) was determined by paired t test and one-way ANOVA. The data are expressed as the means ± S.E.Ms. or as percentages of the control values. The effect of GP handling on the RTn firing patterns was analyzed by the burst index (BI), which was calculated by dividing ISIs < 10 ms by ISIs < 200 ms. The power data were normalized and expressed as the means between 5 and 50 Hz. The coherence was considered significant upward of 95% of the confidence limit [49–51].

## Results

### Firing characteristics of RTn neurons and localization.

**Fig. 1:**
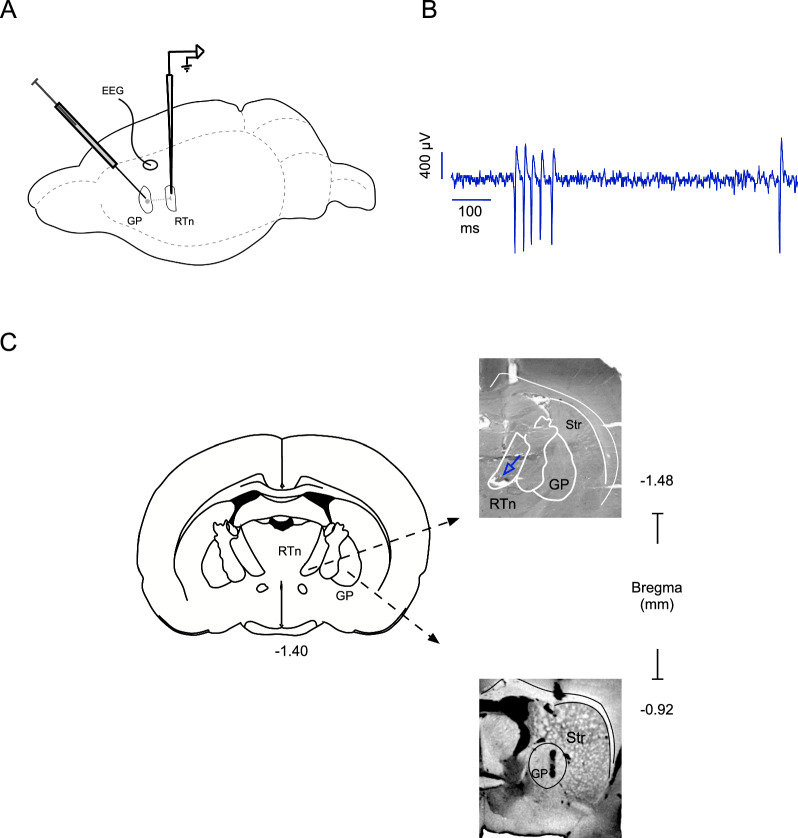
Experimental procedure and histological localization of RTn neuron recordings. **A** Schematic representation of the experimental setup, which included pharmacological stimulation of the GP, extracellular unit recording of RTn neurons, and EEG recordings of the motor cortex. **B** Raw traces of firing patterns characteristic of RTn neuron recordings. **C** The image shows histological verification of the recording (upper) and microinjection (down) zones

In this study, all neurons recorded in the RTn were localized to the rostral portion and showed an interchange between tonic and burst firing (irregular firing pattern). The total number of recorded neurons was 97 (mean spiking frequency = 7.29 ± 0.73 spikes/s, and the mean BI was 0.55 ± 0.044; Fig. 1).

### Response of RTn neurons to activation of the GABA B-Rs in the GP

**Fig. 2:**
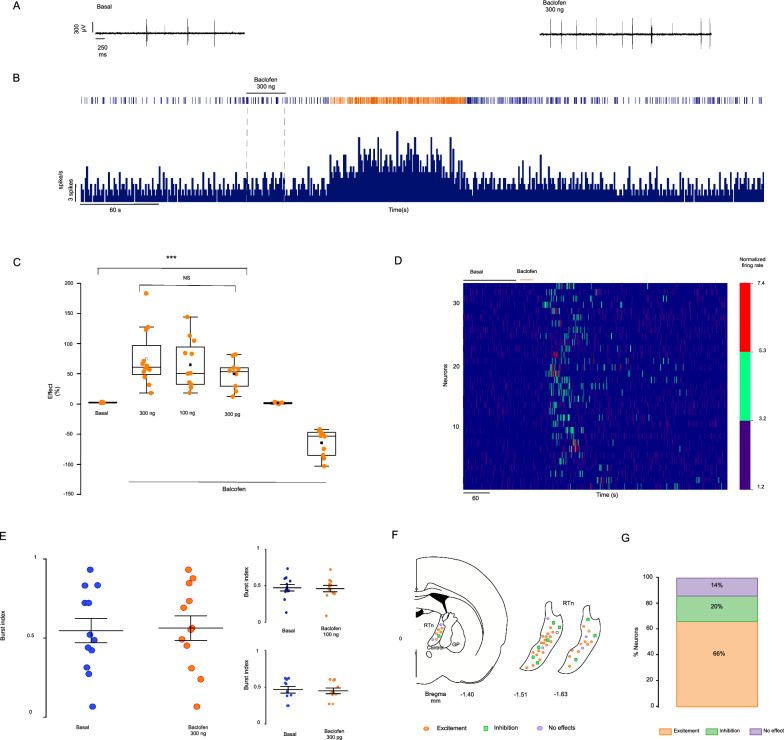
The activation of GABA B-Rs in the GP increases the firing rate of RTn neurons. **A** Sample of raw traces of the basal (left) firing activity of RTn neurons and their firing activity after (right) microinjection of GABA B agonist baclofen into the GP. The voltage and time scales apply to both traces. **B** Peri-event histogram and raster representation of the spiking activity of RTn neurons after administration of 300 ng of baclofen into the GP. The black horizontal segment and dashed line represent the injection period. Both graphs are of the same neuron. **C** Statistical analysis the effect of the application of different concentrations of baclofen to the ipsilateral GP on the RTn neuron firing rate. The effect is expressed as percent changes from the basal activity. No significant difference was observed between all doses applied to the GP (p = 0.5511; one-way ANOVA). Ten neurons showed a decreased firing rate after baclofen treatment (p = 0.00034, paired t test), and seven neurons showed no response to any concentration (p = 0.056; paired t test). Each circle represents one neuron—NS: not significant; *** p < 0.05. **D** Heatmap of the normalized (z score for the mean) firing rate of all neurons recorded before and after baclofen application into the GP. All doses of baclofen tested are represented. The orange horizontal bar represents the injection period. **E** Statistical analysis of the effects of all doses of baclofen on the burst index compared with the basal (300 ng: p = 0.50, paired t test; n = 12 neurons. 100 ng: p = 0.20, paired t test; n = 12 neurons; 300 pg: p = 0.27, paired t test; n = 10 neurons.). Each symbol represents one neuron. **F** Representation in the coronal plane of the recording sites and type of response of RTn neurons. **G** The graph represents the type of response of RTn neurons as a percentage

Different doses of GABA B-R agonists were applied to the GP to determine the effect of GABA B-Rs on the RTn neuron firing rate. All doses of baclofen evoked an enhancement in the spiking frequency of RTn neurons (Fig. 2). The first dose evaluated was 300 ng. This dose increased the firing frequency by 74.66 ± 13.50% relative to the basal values (mean basal firing rate = 9.38 ± 2.04; mean firing rate after baclofen administration = 14.16 ± 2.71; p = 0.0008, paired t test; n = 12 neurons). The second tested dose (100 ng) exerted the same effect but with a smaller magnitude: the spiking was enhanced by 64.72 ± 11.62% (mean basal firing rate = 5.23 ± 1.62; mean firing rate after baclofen administration = 8.89 ± 2.44; p = 0.00192, paired t test; n = 12 neurons). The last dose evaluated was 300 pg, and this dose increased the firing rate by 49.82 ± 7.28% (basal mean firing rate = 9.05 ± 1.49; mean firing rate after baclofen administration = 13.35 ± 2.34; p = 0.00286, paired t test; n = 10 neurons). However, there was no significant difference between all doses applied to the GP (p = 0.5511; one-way ANOVA, [Fig. 2C]). The effect had a mean duration of 106.6 s. The firing pattern was evaluated in the RTn neurons showing a significant percentage response (mean basal BI = 0.55 ± 0.08; basal BI after a baclofen dose of 300 ng = 0.57 ± 0.08; p = 0.50, paired t test; n = 12 neurons; Fig. 2E left. BI with 100 ng of baclofen; basal 0.47 ± 0.044; baclofen 0.45 ± 0.043; p = 0.20, paired t test; n = 12 neurons; Fig. 2E right-top. BI with 300 pg of baclofen; basal 0.49 ± 0.0034; baclofen 0.48 ± 0.029; p = 0.27; paired t test; n = 10 neurons; Fig. 2E right-bottom), and the CV remained unchanged. Ten neurons showed a decreased firing rate by 41.10 ± 8.12% after baclofen treatment (mean basal firing rate = 16.98 ± 3; mean firing rate after baclofen administration = 11.98 ± 2.20; p = 0.00034, paired t test; n = 10 neurons; Fig. 2C), and seven neurons showed no response to any concentration (basal mean spiking activity = 4.31 ± 1.80; mean spiking activity after baclofen administration = 4.09 ± 1.84; p = 0.056; paired t test; n = 7 neurons; Fig. 2C). The location of each neuron's recording is shown in Fig. 2F.

In the control experiment, 100 nl of NaCl solution (0.9% w/v) was applied to the GP. Five neurons recorded after application of the NaCl solution did not show a change in their frequency discharge or firing pattern (spiking rate = 8.94 ± 1.14 spikes/s [basal] vs. 8.9 ± 1.18 spikes/s [NaCl]; BI: 0.47 ± 0.04 [basal] vs. 0.45 ± 0.028 [NaCl]; CV: 0.40 ± 0.18 [basal] vs. 0.44 ± 0.19 [NaCl]; p = 0.35; t test; n = 5 neurons).

### Effect of GABA B-Rs on the spiking rate of GP neurons.

**Fig. 3:**
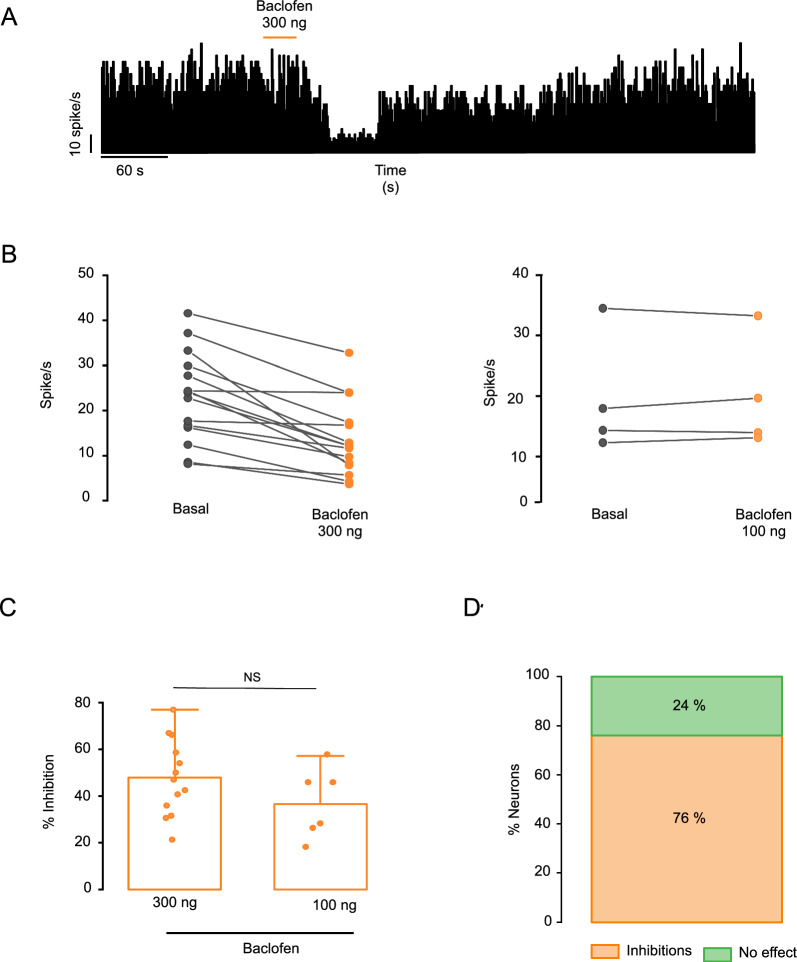
Effects of GABA B activation on GP neurons. **A** Histogram representation of one GP neuron spiking activity after the local administration of 300 ng of baclofen. The orange horizontal line represents the injection period. **B** Left. Spiking rate of GP neurons under basal conditions and effects after pharmacological stimulations with 300 ng of baclofen. The firing rate of GP neurons decreased relative to the basal values after the local injection of baclofen (p = 0.00003; paired t test; n = 13 neurons). Right. Spiking rate of GP neurons both in the basal condition and after pharmacological stimulations with 100 ng of baclofen. Four neurons showed no change in firing rate in response to this concentration of baclofen (p = 0.74; paired t test; n = 4 neurons). **C** Statistical analysis of the effect of the application of different concentrations of baclofen to the GP. The effects are expressed as percent changes relative to the basal activity. No significant difference was observed between the doses applied to the GP (p = 0.2577; two-tailed t test). **D** The graph represents the response of GP neurons as a percentage

The firing rate of GP neurons decreases after application of baclofen (Fig. 3). Two concentrations of baclofen were applied: 300 ng and 100 ng. The baclofen concentration of 300 ng reduced the firing rate of GP neurons by 47.85 ± 4.53 (basal mean firing rate = 23.30 ± 2.97, mean firing rate after baclofen = 12.38 ± 2.29; p = 0.00003; paired t test; n = 13 neurons; Fig. 3B left-C). Two neurons did not respond to this concentration (basal spiking rate = 20.99 ± 3.37, mean spiking rate after baclofen = 20.36 ± 3.68; p = 0.28; paired t test; n = 2 neurons). The baclofen concentration of 100 ng reduced the firing rate by 36.65 ± 6.08% in six pallidal neuron recordings (basal mean rate = 11.38 ± 2.65, mean rate after baclofen = 7.96 ± 2.40. Figure 3C). Four neurons showed no response to this concentration of baclofen (basal mean firing rate = 18.49 ± 5.06, mean firing rate after baclofen = 18.69 ± 4.70; p = 0.74; paired t test; n = 4 neurons. Figure 3B right). Similar to the results found from analyzing the responses of RTn neurons, no significant difference was found between the tested doses (p = 0.2577; two-tailed t test; Fig. 3C).

### MCx activity in response to a GABA B-R agonist in the GP and its effect on the RTn

**Fig. 4:**
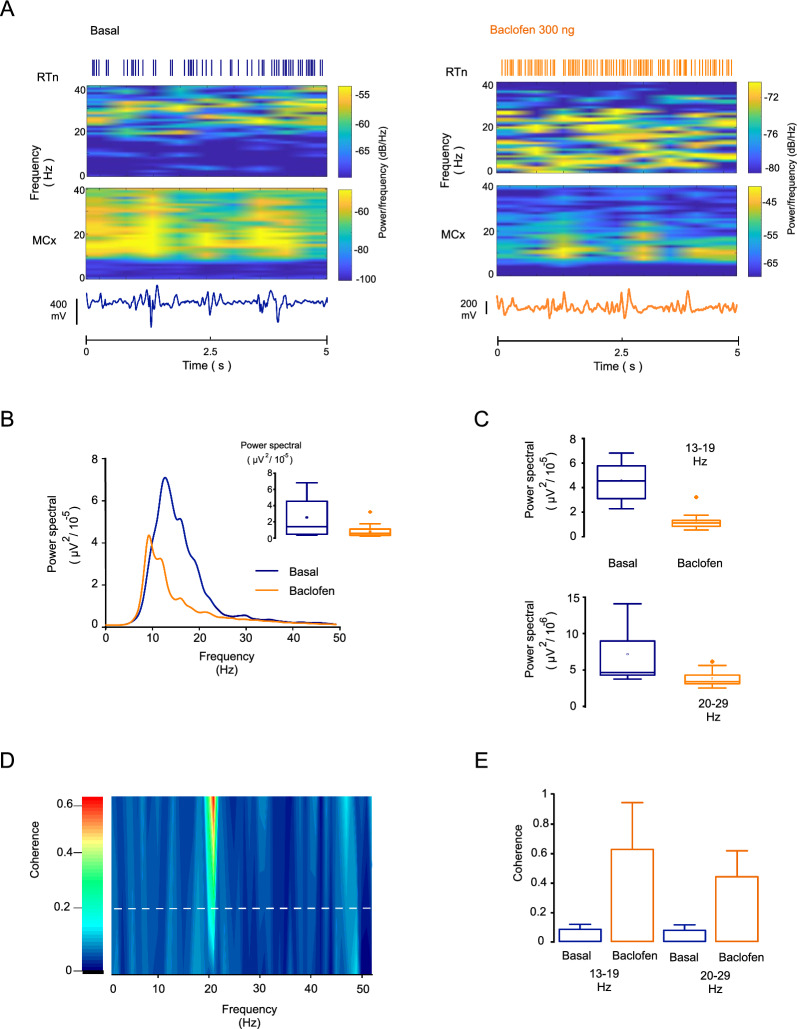
Enhancement of the RTn firing rate later GABA B-Rs agonism in GP decreases the cortical beta frequency band. **A** Spectrograms of the RTn neuron and cortical activity under both experimental conditions (5-s bin). Activation of GABA B-Rs in the GP by baclofen (300 ng) injections reduced the spectral density of the cortical beta band and subsequently increased the RTn neuron spiking activity. Top: Raster plot showing the RTn neuron activity under basal conditions and after pharmacological stimulation. The bottom traces illustrate the cortical activity under both basal and baclofen conditions. The time scales apply to both traces. The plot was derived from the same neuron. **B** The graph shows the average power spectrum density under the experimental conditions. Stimulation of GABA B-Rs in the GP with 300 ng of baclofen decreased the spectral density at frequencies of 13 to 30 Hz. The inset plot shows the statistics of the effect of baclofen in the whole beta band. **C** Top: statistics of the effect of the application of baclofen to the ipsilateral GP in the low beta band (13–19 Hz; p = 0.000017; paired t test; n = 13 neurons). Bottom: Statistics of the response to intrapallidal baclofen in the high beta band (20–30 Hz; p = 0.007; paired t test; n = 13 neurons). **D** Heatmap showing the higher coherence between the MCx and RTn neurons in the beta frequency band. Coherence showed a significant increment relative to the basal values after baclofen administration (mean basal = 0.082 ± 0.006, mean after baclofen = 0.62 ± 0.06; p = 0.000007, paired t test; n = 13 neurons). The dashed lines indicate 95% confidence intervals. **E** Graph showing the coherence values after baclofen (300 ng) application between the MCx and RTn neurons at frequencies associated with low and high beta activity. At low beta activity (13–19 Hz), the coherence between RTn and MCx showed a significant increment (mean basal = 0.082 ± 0.006, mean baclofen = 0.62 ± 0.06; p = 0.000007, paired t test; n = 13 neurons). At high beta activity (20–30-Hz), coherence increased significantly relative to the basal values (basal = 0.07 ± 0.006, mean baclofen = 0.43 ± 0.03; p = 0.000002; paired t test; n = 13 neurons; **E**)

The increment in the spiking rate of the RTn after agonism of GABA B-Rs in the GP decreased the power spectra in the beta frequency in the MCx (Fig. 4). During simultaneous recordings of the RTn and MCx activity, the basal firing rate of the RTn was adjusted to a frequency of 20.73 Hz (range = 17–26 Hz, Fig. 4A top), and the frequency of MCx basal activity was set at a frequency of 19.25 Hz (range = 11–24 Hz, Fig. 4A). The RTn firing enhancement induced by agonism (300 ng of baclofen) of pallidal GABA B-Rs was adjusted to a frequency of 26.11Hz (range = 22–29 Hz), and the effect on MCx activity was set at a frequency of 11.65 Hz (range = 10–16 Hz; Fig. 4A bottom). The power spectral density of the MCx after baclofen administration was 55.74 ± 4.78% compared with the basal values (basal mean power = 2.53 × 10^–5^ ± 5.28 × 10^–6^ µV^2^, mean power after baclofen [300 ng] administration = 8.04 × 10^–6^ ± 1.65 × 10^–6^ µV^2^; paired t test p = 0.0003; n = 13 neurons; Fig. 4B).

An analysis of low (13–19 Hz) and high (20–30 Hz) beta bands showed a significant decrease in the power density in both bands (Fig. 4C). The magnitude of the power reduction in the low beta band was 73.30 ± 2.64% (mean basal power = 4.58 × 10^–5^ ± 5.55 × 10^–6^ μV^2^; mean power after the administration of 300 ng of baclofen = 1.28 × 10^–5^ ± 2.71 × 10^–6^ μV^2^; paired t test, p = 0.000017; n = 13 neurons; Fig. 4C top). The percent reduction in the high beta band was 39.62 ± 5.34% (basal power = 7.13 × 10^–6 ^± 1.33 × 10^–6^ μV^2^, power after baclofen administration = 3.85 × 10^–6^ ± 4.24 × 10^–7^ μV^2^; paired t test p = 0.007; n = 13 neurons; Fig. 4C bottom).

The coherence at the beta band was higher between RTn and MCx activity after the activation of GABA B-Rs into the GP (Fig. 4D). At 13–19 Hz, the coherence between the increase in the firing of RTn neurons by agonism of GABA B-Rs into the GP showed a significant increment relative to the basal values (mean basal = 0.082 ± 0.006, mean after baclofen administration = 0.62 ± 0.06; p = 0.000007, paired t test; n = 13 neurons). Similarly, the 20–30-Hz band exhibited a higher coherence relative to the basal values (basal = 0.07 ± 0.006, mean after the administration of 300 ng of baclofen = 0.43 ± 0.03; p = 0.000002; paired t test; n = 13 neurons; Fig. 4E).

### Response of RTn neurons to antagonism of GABA B-Rs in the GP

**Fig. 5:**
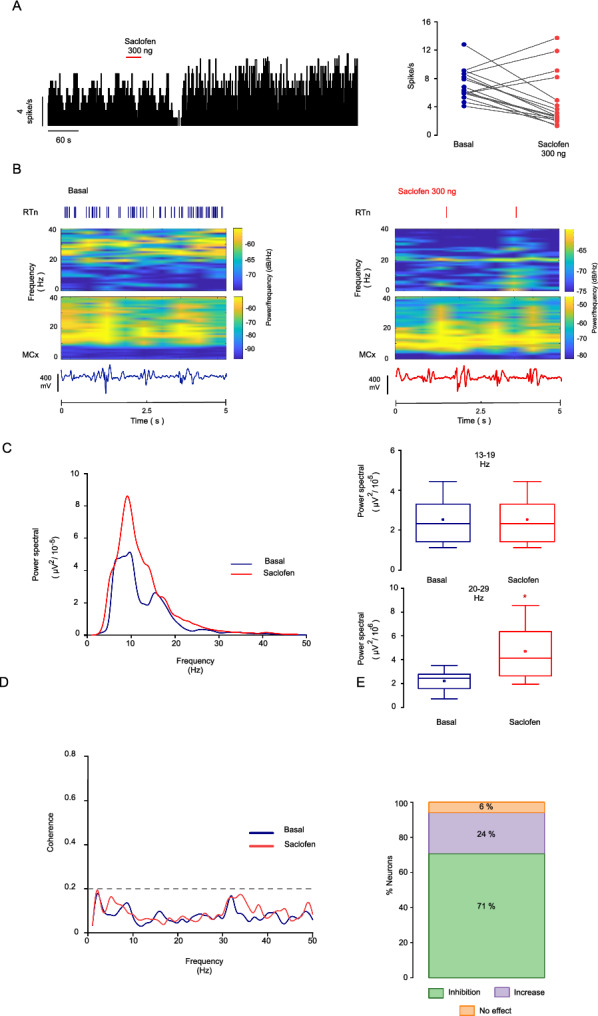
Antagonism of GABA B-Rs in the GP inhibits the activity of RTn neurons and the reduction in the power spectra in the cortical beta band. **A** Histogram of one RTn neuron firing activity after GP administration of saclofen (300 ng). The red line represents the injection period. Right: Spiking rate of RTn neurons under basal conditions and after pharmacological stimulation with 300 ng of saclofen. The firing rate of RTn cells decreased relative to the basal values after local injection of saclofen; p = 0.000001, paired t test; n = 12 neurons). The same doses of saclofen increased the spiking activity in four neurons (basal = 7.12 ± 0.94 spikes/s, saclofen = 10.75 ± 1.37 spike/s; p = 0.005, paired t test, n = 4 neurons). **B** Spectrograms of the RTn and cortical activity under experimental conditions (5-s bin). The blockade of GABA B-Rs by 300 ng of saclofen in the GP did not modify the spectral density of the cortical beta band after reducing the firing rate of RTn neurons. Top: Raster plot representing the RTn neuron activity in the basal state and after pharmacological blockade. Bottom: Traces illustrating the cortical activity under both basal and baclofen conditions. The time scales apply to both traces. The plot was derived from the same neuron. **C** Left: The plot shows the average power spectral density under the experimental conditions. The blockade of GABA B-Rs in the GP with 300 ng of saclofen does not modify the spectral density in the 13–19Hz frequency. However, an increase in the power spectral density is observed in the frequency range of 20–30 Hz. Right: Statistics on the effect of the application of saclofen to the ipsilateral GP at 13–19 Hz (p = 1, paired t test; n = 12 neurons, top) and 20–30 Hz (p = 0.00365, paired t test; n = 12 neurons, bottom). **D** Left: Graph showing a lack of coherent activity at frequencies associated with the beta band between the MCx and RTn neuron activity. The dashed lines represent the 95% confidence intervals. Right: The graph represents the percent response of the RTn neurons after antagonism of GABA B-Rs in the GP

The effect of saclofen (a highly potent and selective antagonist of GABA B-Rs) was evaluated to confirm the involvement of GABA B-Rs in RTn neuron activity. The infusion of 300 ng of saclofen decreased the spiking activity of RTn neurons to 69.86 ± 3.23% (basal = 6.86 ± 0.74 spikes/s, after saclofen = 2.09 ± 0.33 spikes/s; p = 0.000001, paired t test; n = 12 neurons; Fig. 5A). Four neurons showed an enhancement in spiking activity after the same doses of saclofen (basal = 7.12 ± 0.94 spikes/s, saclofen = 10.75 ± 1.37 spike/s; p = 0.005, paired t test, n = 4 neurons), and one neuron did not exhibit a response. Under basal conditions, the spontaneous firing of the RTn was located at a frequency of 22.15 Hz (range 19–26 Hz, Fig. 5B top), and the cortical activity was located at 20.60 Hz (range 19–24 Hz, Fig. 5B top). After GABA B antagonism in the GP, the reduction in the firing activity of the RTn was located at a frequency of 13.67 Hz (range = 9.66–19.24 Hz), and the cortical oscillations were located at a frequency of 21.31 Hz (range = 15–24 Hz, Fig. 5B bottom).

The power spectral density of the MCx did not show a change after the decrease in the firing activity of the RTn induced by antagonism of GABA B-Rs in the GP. The power spectral density in the low beta band was similar to the basal values (basal power spectral density = 2.52 × 10^–5^ ± 4.17 10^–6^ μV^2^, power spectral density after saclofen administration = 2.52 × 10^–5^ ± 4.17 10^–6^ μV^2^; p = 1, paired t test; n = 12 neurons, Fig. 5C). Nevertheless, the high beta band in the power spectra increased relative to the basal values in response to decreases in the RTn spiking activity induced by intrapallidal injection of saclofen (basal power density = 2.76 × 10^–5 ^± 2.94 × 10^–7^ μV^2^, power density after saclofen administration = 5.27 × 10^–6^ ± 7.77 × 10^–7^ μV^2^; p = 0.00365, paired t test; n = 12 neurons). The GABA B-R blockade in the GP did not exhibit a coherent response between the RTn and MCx (Fig. 5D left).

**Fig. 6:**
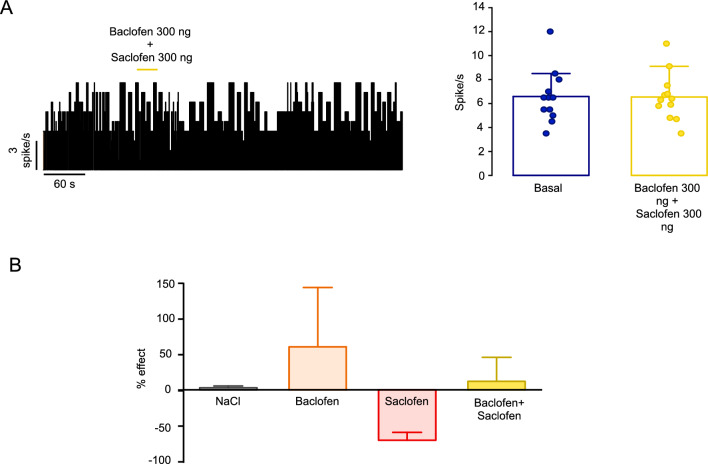
The coadministration of baclofen and saclofen to the GP has no effects on RTn neurons. **A** Left: Frequency histogram of one RTn neuron firing activity after coadministration of baclofen (300 ng) and saclofen (300 ng) in the GP. The horizontal yellow line represents the injection period. Right: Spiking rate of RTn neurons under basal conditions and after pharmacological stimulation by coadministration of baclofen and saclofen. The firing rate of RTn cells after local coinjection did not differ from the basal values; p = 0.79, paired t test; n = 12 neurons). **B** The plot summarizes the response of the RTn under all application conditions. The effects are expressed as percent changes relative to the basal activity

The effects of the coadministration of baclofen and saclofen were evaluated in another series of trials. The coadministration in the GP (300 ng baclofen + 300 ng saclofen) did not increase the spiking rate of 12 RTn neurons (basal = 6.58 ± 0.63 spike/s, baclofen + saclofen = 6.54 ± 0.57 spike/s; p = 0.79, paired t test) (Fig. 6). The effect of the RTn under all application conditions is summarized in Figure 6B.

## Discussion

Important lines of evidence emerge from our results: tonic modulation of the spontaneous activity of the RTn and by GP mediated by GABA B-Rs, functional role of the GP-RTn pathway in the BG-Th-Cx network at the beta frequency, and participation of GABA B-Rs in circuit function.

The integrative role of the GP in BG physiology involves GABAergic transmission to diverse areas and its firing rate [1]. In these functions, the GP firing is inhibited by GABA B-Rs [35–38], and its projections control the spontaneous activity of the RTn [42, 52], similarly, inhibition of the GP increases the spiking activity of RTn neurons [42]. Based on this evidence, we evaluated the spiking activity of the RTn after pharmacological activation of pallidal GABA B-Rs. This study revealed higher spontaneous activity of RTn neurons after pallidal infusion of baclofen. We correlated the activation of the GABA B-Rs of the GP with the activation of RTn neuron spiking because we found inhibition of GP firing after baclofen administration (similar to pioneering results [35–38]), and this response and the effect on RTn neurons were reversed by coadministration in the GP with the more highly selective antagonist [35–38] saclofen. Given this context, we assume that the inhibition of GP neurons by GABA B-Rs reduces the inhibition of the RTn and increases its firing rate. Another possibility that explains our results is that baclofen infusion did not directly stimulate the neurons that project to the RTn. However, despite the above findings, RTn neurons send collaterals to neighboring neurons that they tonically inhibit and thus generate disinhibition adjacent to the inhibitions [16, 55]. As a result, the registered neurons are disinhibited by inhibiting the neighboring neurons.

The current evidence of the localization and function of GABA B-Rs at the synaptic level in the BG circuit provides a broad mechanism that explains our results. GPs express GABA B-Rs presynaptically at both the striatopallidal (GABAergic) and subthalamic (glutamatergic) terminals [34, 53, 54] and at the postsynaptic level in dendrites. The inhibition of glutamatergic transmission by the activation of presynaptic GABA B-Rs has been demonstrated in several brain areas [39, 56–58], including the BG [59–62] and GP [63]. In this sense, the local application of glutamate increases the GP firing rate and lessens RTn neuron activity [42]. Thus, baclofen increases the spiking rate of the RTn by reducing glutamate release into GP neurons through the activation of presynaptic receptors in subthalamic terminals. Moreover, the activation of presynaptic GABA B-Rs in striatopallidal terminals reduces GABA release and thereby increases spatial and temporal inhibition [37, 64]. [39]. Both events contribute to GP inhibition, which allows the disinhibition of RTn neurons.

Additionally, the GP shows high ambient GABA levels under basal conditions [1] and expression of GABA B-Rs in extrasynaptic regions [65]. Thus, higher GABA levels favor leaking to extrasynaptic sites and the activation of extrasynaptic GABA B-Rs under basal conditions and during phasic synaptic transmission. A similar effect is observed after blockade of GABA transporter type 1 [(GAT-1); [63]]. In this context, the pharmacological elevation of the GABA levels in the GP increases the spiking discharge of the RTn [41]. Previous evidence contributes to our finding of an increase in the spontaneous firing of the RTn by activation of GABA B-Rs in the GP, and we hypothesize that GABA B-Rs are tonically activated and modulate the RTn through the GP-RTn pathway. Accordingly, we provide evidence showing that GABA B-Rs in the GP modulate the functionality of the network, as has been shown in other brain regions [32, 66–69].

In contrast, we identified a group of RTn neurons that showed no decrease in firing activity after baclofen administration and another group of neurons that showed no response to any concentration of baclofen. GP neurons show morphological and functional heterogeneity [1, 7, 70, 71]. As part of this heterogeneity, a baclofen-insensitive neuronal subpopulation has been described [72]. This observation indicates that a group of GP neurons do not show a response to the administration of baclofen and consequently does not affect RTn neuron firing. Lateral inhibition is accepted as a functional mechanism in the interaction of collaterals within the GP [73]. This process provides other evidence explaining the increase in the activity of the RTn neurons. When a GP neuron is inhibited, it releases the neurons that receive its collateral, and thus, the released neuron increases its firing, which inhibits the RTn neuron.

In the RTn, regional diversity in the firing pattern is accepted as an essential functional characteristic [74, 75]. In the neural network context, the transition in the firing mode shapes the information output that leads to the physiological state. Similarly, it has been suggested that tonic inhibition contributes to rapid modification of synaptic integration in a cell population and thus modulates the neuronal output pattern [76]. Our study focused on neurons with an irregular firing pattern,in this framework, we described an increase in the mean spiking rate induced by GABA B-R activation in the GP without a change in the spiking mode in these neurons. A similar effect was previously observed in this type of neuron after administration of GABA into the GP [42] and secondary to the increase in GABA levels in the GP after the pharmacological blockade of GAT-1 [41]. During tonic inhibition, a similar firing mode behavior has been observed in two types of inhibitory neurons of the cerebellar cortex [76]. Based on this evidence, we hypothesize that the GP tonically modulates reticular neurons with irregular firing and thus modulates information transfer to the Th-Cx circuit.

In the GP, GABA B responses are evoked by both striatal and local collateral axon GABA release [38, 54]. During synaptic transmission, the GABA B-Rs activated by GABA release play a part in the feedback and feedforward control of the neurons in the target region [1, 36, 37, 77]. In addition, the modulation of GABA B-Rs has been reported in several brain circuits [78–80]. Similarly, our results are in line with these conclusions because the GP sends axons to the rostral part of the RTn [10, 11]. Additionally, we found that activation of GABA B-Rs in the GP increases the firing rate of RTn neurons; hence, our results suggest the involvement of the GP-RTn pathway in the control of information flow in the BG-Th-Cx network.

We found that the administration of baclofen to the GP reduces the spectral density of the MCx in the beta frequency. This effect showed coherence between the firing activity of the RTn and the MCx at the same frequency band. Our observation was contrary to the effect observed with different conditions. The intraperitoneal application of baclofen increases the power spectral density in the beta band [81, 82]. However, similar effects (a reduction in the power spectral density) were found on gamma oscillations in the hippocampus [78, 81]. Two events allow establishment of a feasible explanation for diminishing the beta power. First, the increase in the spiking frequency of RTn neurons inhibits the burst of TC neurons sent to the cortex [19, 83]. Second, neurons in the rostral zone of the RTn send axons to ventrolateral nuclei (VLs) and the MCx [26, 84, 85]. Therefore, an increase in the spiking frequency of RTn neurons inhibits VL neurons [13, 26]. In light of this functional connectivity, we hypothesize that VL neurons are inhibited by increases in the spiking frequency of the RTn rostral neuron after GABA B-R activation in the GP.

The antagonism of GABA B-Rs in the GP increases its firing rate [1, 36]. This effect is secondary to reducing the activity of GABA B-Rs by the antagonism of both pre-and postsynaptic receptors [27, 86] in the glutamatergic subthalamic terminal. The reduced excitation results in un-inhibition [64]. The above mentioned evidence supports our observation that GP injection of saclofen decreased the firing rate of the RTn. Thus, the disinhibited GP neurons increased their firing rate and thus inhibited neurons in the RTn, and this inhibition had an essential impact on cortical activity. We found that RTn inhibition by the blockage of GABA B-Rs in the GP reverses the effect of baclofen on the power spectral density at 13 to 19 Hz. These results align with the effects of GABA B-Rs on network function: modulation of the spiking activity of individual neurons during oscillation [87]. We also present evidence showing that inhibition of the RTn after injection of the antagonist of GABA B-Rs into the GP increases the power spectral density at 20–29 Hz. Similar results have been found for oscillatory events in other brain areas, although in other frequency bands [88–91]. Based on the above-described results, we hypothesize that the GP, through GABA B-Rs, desynchronize cortical beta oscillations by disinhibiting reticular neurons.

Oscillatory activity in the beta frequency band is prominent in the MCx. Variations in the power density in this frequency band have been linked with the stage of motor activity. The decrease (desynchronization) has been correlated with the onset of movement, unilateral movement of a limb [92], and during ipsilateral execution movements [8, 93]. Our results display evidence of physiological events underlying these results because we observed that a decrease in the ipsilateral beta power was more coherent with an increase in activity in the same frequency band after activation of GABA B-Rs in the GP, which suggests that the GP modulates beta activity during the stages of movement through the GP-RTn pathway. Interestingly, GABA B-Rs have been implicated in ipsilateral pivoting [35, 47], and it has been speculated that the GP-RTn connection is involved in this motor event [47]. Our results provide evidence supporting this hypothesis.

In contrast, frequencies of 11 to 30 Hz are accepted as anti-kinetic; similarly, it has been suggested that desynchronization could reflect a less efficient transition between processing states [94]. Consequently, desynchronization in the beta band is necessary for the initiation of movement, which is favored by disinhibition of the RTn; therefore, the tonic inhibition of the RTn by the GP and subsequent modulation of beta activity may contribute to motor behaviors. However, although our results suggest that the GP-RTn participates in the flow of motor information within the BG-Th-Cx network, future studies are needed to confirm this hypothesis.

Our results conclude that the GP, through GABA B-Rs, modulate the spontaneous firing of RTn neurons tonically; consequently, these receptors decrease the cortical beta power, which suggests that the GP exerts control by disinhibition the RTn and contributes to cortical beta oscillation activity.

## Data Availability

The datasets generated and/or analyzed during the current study are available from the corresponding author upon reasonable request.

## Abbreviations

GP: External globus pallidus
BG: Basal ganglia
GABA B-Rs: GABA B receptors
Th: Thalamus
VL: Ventrolateral nuclei
Cx: Cortex
MCx: Motor cortex
RTn: Thalamic reticular nucleus
GABA: Gamma-aminobutyric acid
EEG: Electroencephalogram
